# scANMF: Prior Knowledge and Graph-Regularized NMF for Accurate Cell Type Annotation in scRNA-seq

**DOI:** 10.3390/ijms27010125

**Published:** 2025-12-22

**Authors:** Weilai Chi, Ying Zheng, Huaying Fang, Shi Shi

**Affiliations:** 1 School of Mathematical Sciences, Capital Normal University, Beijing 100048, China; zy18851900972@163.com; 2 Beijing Advanced Innovation Center for Imaging Theory and Technology, Capital Normal University, Beijing 100089, China; 3 Academy for Multidisciplinary Studies, Capital Normal University, Beijing 100089, China; 4 School of Economics, Hangzhou Dianzi University, Hangzhou 310018, China

**Keywords:** single-cell RNA sequencing, cell-type annotation, non-negative matrix factorization, prior knowledge integration, graph regularization, robustness to noise, semi-supervised learning

## Abstract

Single-cell RNA sequencing (scRNA-seq) provides a high-resolution view of cellular heterogeneity, yet accurate cell-type annotation remains challenging due to data sparsity, technical noise, and variability across tissues, platforms, and species. Many existing annotation tools depend on a single form of prior knowledge, such as marker genes or reference profiles, which can limit performance when these resources are incomplete or inconsistent. Here, we present scANMF, a prior- and graph-regularized non-negative matrix factorization framework that integrates marker-gene information, partial label supervision, and the local manifold structure into a unified annotation model. scANMF factorizes the expression matrix into interpretable gene–factor and cell–factor representations, enabling accurate annotation in settings with limited or noisy prior information. Across multiple real scRNA-seq collections, scANMF achieved a high annotation accuracy in within-dataset, cross-platform, and cross-species evaluations. The method remained stable under varying levels of label sparsity and marker-gene noise and showed a broad robustness to hyperparameter choices. Ablation analyses indicated that marker priors, label supervision, and graph regularization contribute complementary information to the overall performance. These results support scANMF as a practical and robust framework for single-cell annotation, particularly in applications where high-quality prior knowledge is restricted.

## 1. Introduction

Cells are the fundamental structural and functional units of biological systems, displaying a remarkable heterogeneity in gene expression programs and phenotypic states. The accurate delineation of distinct cellular subtypes within complex tissues is essential for understanding their functional contributions to processes such as hematopoiesis, embryonic morphogenesis, and intestinal development [1,2,3,4]. Conventional strategies such as cell sorting and microscopy-based isolation followed by transcriptomic or proteomic profiling have provided important insights. However, they remain constrained by labor-intensive workflows, technical complexity, and reliance on manual processing [5,6,7]. Moreover, bulk RNA sequencing—long the dominant tool for transcriptomic studies—compounds these limitations by averaging the expression across populations, thereby obscuring heterogeneity and masking rare subsets [8].

The emergence of single-cell RNA sequencing (scRNA-seq) has directly addressed these challenges by enabling transcriptomic profiling at a cellular resolution. This transformative approach allows for the deconvolution of complex tissues, the identification of previously unrecognized cell types, and the dynamic characterization of processes such as lineage differentiation and immune responses in disease and development [2,9,10]. Methodological advances have rapidly expanded its scale, evolving from low-throughput protocols to high-throughput droplet-based systems that underpin current large-scale cellular atlas projects [11]. Through these innovations, scRNA-seq has become a cornerstone technology across diverse disciplines, including developmental biology, oncology, and neuroscience [12,13].

The identification of cellular populations in scRNA-seq data plays a vital role in single-cell transcriptomic studies, forming the basis for downstream biological interpretation. Traditionally, it relies on the unsupervised clustering of cells according to their expression profiles, followed by cluster annotation through the use of marker genes that are differentially expressed between clusters [14,15]. To assign cell-type labels, these candidate markers must be cross-validated through an extensive literature review or the consultation of curated cell marker databases. However, this manual curation process is both labor-intensive and error-prone, as marker genes are frequently expressed across multiple clusters and may be associated with more than one cell type [16]. It is thus imperative to establish automated approaches capable of accurately classifying single cells without reliance on manual marker gene selection. The current computational paradigms for automated cell annotation predominantly fall into two categories: methods that leverage predefined marker gene sets and those that employ fully annotated reference datasets [17].

Marker-based strategies, which leverage prior knowledge of cell-type-specific genes, are generally built on two main principles: probabilistic modeling and scoring-based annotation. Methods such as SCINA [18], CellAssign [19], and scSorter [20] exemplify probabilistic approaches, employing statistical frameworks to assign cells to known or novel types while accounting for noise and variability. In contrast, methods such as scCATCH [21], scMRMA [22], and ScType [16] represent scoring-based strategies, aligning clusters to reference databases or marker gene signatures through evidence-based or enrichment scoring schemes. In addition, multiple databases specifically designed for scRNA-seq data—such as PanglaoDB [23,24], CellMatch [21,25], SCsig [26], and CellMarker [27,28]—provide extensive resources linking cell types to their corresponding marker genes. However, these approaches remain constrained by the completeness and specificity of existing marker databases, exhibit a limited ability to discover novel cell types, and are often highly dependent on the accuracy of prior clustering results.

In contrast, reference-based annotation methods infer cell identities by comparing scRNA-seq data with well-annotated reference datasets, generally following two main principles: correlation-based matching and supervised classification. Correlation-based approaches, such as scmap [29], CHETAH [30], OnClass [31], SingleR [32], and Symphony [33], evaluate similarities between query cells and reference profiles, often through nearest-neighbor searches, hierarchical assignments, or enrichment scoring, to predict the most likely cell types. In contrast, supervised methods, including scPred [34], SingleCellNet [35], scAnnotate [36], SciBet [37], and more recent methods such as PCLDA [38] and scSorterDL [39], employ machine learning classifiers trained on annotated datasets to capture expression patterns and transfer labels to new data, thereby improving the prediction accuracy and generalizability across platforms. Also, there are methods based on semi-supervised learning that leverage both labeled and unlabeled data to improve the annotation accuracy, such as HiCat [40]. In parallel, graph neural network (GNN)-based approaches such as scGCN [41] propagate label information over the cell–cell similarity graph to learn topology-aware representations, providing another direction for supervised or semi-supervised annotation. The advent of large-scale projects such as the Human Cell Atlas [42,43], Tabula Muris [44,45], and the Mouse Cell Atlas [46,47] has greatly expanded the utility of reference-based strategies by providing comprehensive cross-tissue and cross-species resources. Nevertheless, the effectiveness of these methods is still highly contingent on the quality, representativeness, and curation of reference data. Although they enhance the reproducibility and scalability, their performance remains limited by incomplete or biased references and is further confounded by technical artifacts such as batch effects and inaccurate reference labels. Anchor-based reference integration frameworks, such as the Seurat label transfer framework [48] and its Azimuth pipeline [49], combine shared latent representations, curated reference atlases, and neighborhood information to perform cross-dataset annotation. Although these pipelines improve the consistency through standardized preprocessing and anchor-based mapping, their performance still fundamentally depends on the quality and completeness of the reference atlas.

**Figure 1:**
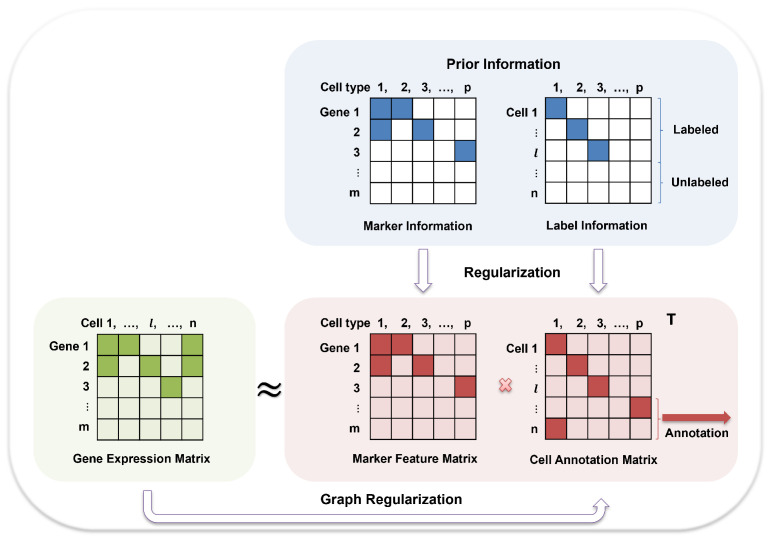
Schematic illustration of the scANMF framework. The model integrates gene expression data with marker and partial label information, factorizing the expression matrix into a marker feature matrix and a cell annotation matrix. The resulting annotation matrix yields accurate and interpretable cell-type assignments.

Notwithstanding these advances, the identification of cell types remains fraught with computational challenges, thus placing substantial demands on analytical frameworks. Reliance on a single form of prior knowledge, whether marker genes or reference datasets, may suffice for well-curated data, but proves inadequate in real-world scenarios. Recent attempts such as scSHAPR [50] integrate marker- and reference-based annotations, but their reliance on multiple external algorithms increases the complexity and restricts the scalability. To overcome these limitations, we propose scANMF, a unified framework that integrates multiple sources of prior knowledge with local geometric constraints into a non-negative matrix factorization model. Specifically, scANMF combines marker gene guidance, partial label information, and graph regularization to simultaneously capture cell-type-specific features and preserve intrinsic cellular geometry. By unifying these components into a single optimization objective, scANMF decomposes the gene expression matrix into interpretable factors, namely a gene feature matrix that captures cell-type-specific signatures and a cell annotation weight matrix that assigns cells to their most likely types. By jointly optimizing these complementary constraints, the model achieves accurate annotation even under noisy or incomplete priors. Moreover, the factorization yields interpretable representations that link inferred factors to biological markers. An overview of the framework is presented in Figure 1.

## 2. Results

### 2.1. Real-Data Analysis

To assess the practical performance of scANMF, we conducted a series of evaluations across three representative real-data scenarios: (i) within-dataset annotation with highly limited labels, (ii) cross-platform annotation across heterogeneous sequencing technologies, and (iii) cross-species annotation, where transcriptional differences are substantial. These analyses collectively examined model behavior under realistic sources of variation in single-cell datasets.

#### 2.1.1. Within-Dataset Annotation

**Figure 2:**
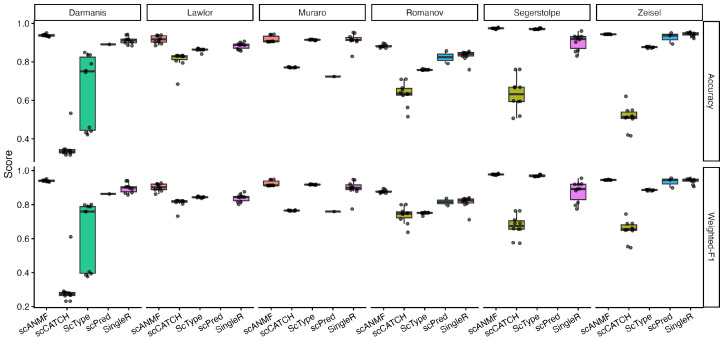
Annotation performance of five methods on real scRNA-seq datasets. scANMF consistently achieved the highest scores across all datasets.

To evaluate the annotation performance under realistic conditions where only a very small subset of cell labels within a dataset can be experimentally identified, we conducted a semi-supervised labeling experiment on pancreas and brain datasets. In each run, we randomly sampled only a minimal number of labeled cells and provided them to scANMF, SingleR, and scPred. In contrast, ScType and scCATCH were applied directly to the remaining unlabeled cells, as they rely solely on predefined marker genes and do not require reference labels. The comparative performance is summarized in Figure 2.

Across all six datasets, scANMF consistently achieved the highest or near-highest accuracy and weighted F1-scores, exhibiting both a superior performance and greater stability across runs. SingleR generally ranked second, followed by ScType, except on the Segerstolpe dataset, where ScType performed better. scCATCH showed a weaker overall performance, particularly on the Darmanis dataset, where ScType also performed poorly. scPred produced valid results in only one to three out of ten runs—and failed entirely on the Lawlor and Segerstolpe datasets—because it trains a separate classifier for each cell type, and thus requires substantially more labeled samples than were available. Nevertheless, in the cases where scPred did produce output, its weighted F1-scores were often higher than its accuracy, a pattern that reappeared in later experiments. Overall, these results indicate that scANMF maintains a stable annotation performance under sparsely labeled conditions across multiple datasets.

#### 2.1.2. Cross-Platform Annotation

We next evaluated the cross-platform performance using two brain datasets generated with distinct protocols (Romanov: Smart-seq2; Zeisel: UMI). Annotation was performed in both directions. As summarized in Table 1, scANMF achieved the highest overall accuracy and weighted F1-scores in both settings. SingleR showed a strong performance when predicting Zeisel, but achieved a lower accuracy when Romanov was the test set. scCATCH and scPred obtained a lower accuracy in both directions, while ScType showed an intermediate performance.

The Romanov dataset contained Ependymal cells that were absent from Zeisel. While reference-based methods such as SingleR suffered a marked drop in accuracy when predicting Romanov, scANMF was able to isolate most of these Ependymal cells. This illustrates that scANMF can identify and separate cell types not present in the training set, underscoring its robustness in situations with incomplete or non-overlapping cell-type coverage across datasets.

We then assessed the performance on pancreas datasets generated by heterogeneous platforms. The Baron–Muraro dataset (10,600 cells) and the Xin–Segerstolpe–Lawlor dataset (4218 cells) were used as training and test sets in both directions. As shown in Table 2, scANMF obtained the highest accuracy and weighted F1 in both settings. SingleR and ScType achieved a slightly lower, but comparable, performance, while scCATCH and scPred showed a reduced accuracy across both directions. We also analyzed the cell-type-specific annotation performance for Pancreas 1, as summarized in Table S3 and Figure S3.

#### 2.1.3. Cross-Species Annotation

We next performed cross-species annotation using human and mouse brain datasets. scCATCH and ScType yielded identical results across the trials because they rely solely on marker genes. scANMF achieved the highest or near-highest accuracy in all four transfer directions across the Zeisel, Romanov, and Darmanis datasets (Table 3). SingleR performed competitively on several tasks, while scCATCH persistently produced lower scores. scPred produced a high weighted F1-score in some settings, but showed a reduced accuracy in others.

Compared with the cross-platform experiments, both scANMF and SingleR remained relatively stable on the Zeisel–Darmanis task, exhibiting only a mild performance decline. However, when using Darmanis to annotate Romanov, the performance of all reference-based methods dropped substantially. This pronounced asymmetry indicates that cross-species differences strongly affect the annotation accuracy, particularly when the cell-type compositions of the training and test datasets are not well aligned.

In addition to the brain data, we further evaluated cross-species annotation using the human and mouse pancreatic datasets from the Baron study. As shown in Table 4, scANMF achieved the best performance in both directions. ScType also performed well, whereas SingleR and scPred showed marked asymmetry between the two directions. In particular, SingleR suffered a substantial decline when annotating human cells using mouse references, and scCATCH performed poorly in both cases. These results indicate that, while species-related differences in transcriptional profiles pose a substantial challenge, scANMF remains robust and consistently preserves class separability across species.

### 2.2. Latent Factors Accurately Recapitulate Cell-Type Marker Structure

**Figure 3:**
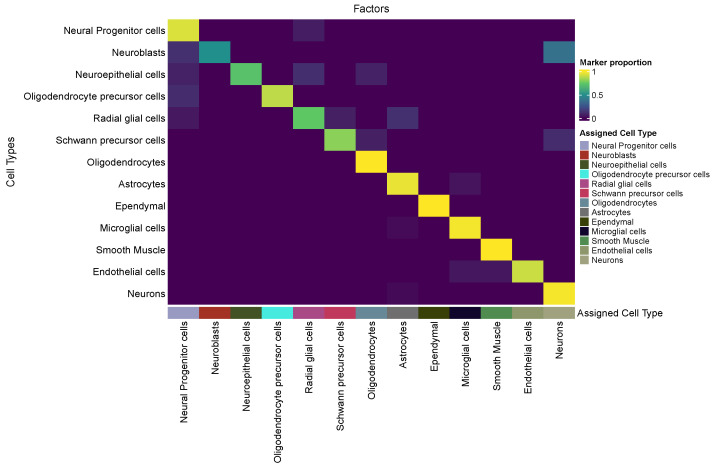
Cell-type-normalized marker-gene proportion matrix $P_{c\to k}$ in the Romanov→Darmanis analysis. Each row denotes a cell type and each column a latent factor. The strong diagonal pattern indicates that each latent factor selectively concentrated the marker genes of a single cell type. The color bar below the heatmap shows the inferred cell-type identity of each factor. This one-to-one correspondence is further supported by a perfect marker-gene accuracy (${\mathrm{Accuracy}}_{\mathrm{marker}}=1.0$), indicating that every marker gene is mapped to a factor consistent with its annotated cell type(s).

We assessed the biological coherence of the latent factors by examining their agreement with marker-gene annotations on the Romanov → Darmanis brain dataset. The cell-type-normalized marker-gene proportion $P_{c\to k}$ revealed a strikingly diagonal structure (Figure 3), indicating that each cell type concentrates nearly all of its marker genes onto a single latent factor. This behavior reflects a near one-to-one mapping between factors and biological cell types. Also, every marker gene was assigned to a factor consistent with at least one of its annotated cell types, yielding a perfect ${\mathrm{Accuracy}}_{\mathrm{marker}}=1.0$. Together, these results demonstrate that scANMF successfully disentangles the transcriptomic space into biologically coherent, cell-type-specific latent factors. A parallel analysis using a pancreas dataset is provided in Supplementary Figure S1, demonstrating the same diagonal structure and high marker-gene consistency.

### 2.3. Robustness Tests and Ablation Studies of scANMF

#### 2.3.1. Robustness Under Noisy Prior Knowledge

**Figure 4:**
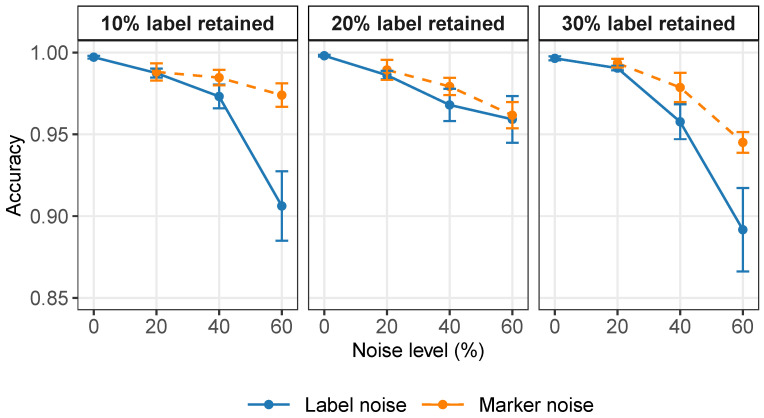
Accuracy of scANMF under different levels of noisy or incomplete prior information. Error bars indicate standard errors across replicates.

Because real-world annotation often suffers from incomplete or inaccurate prior information, we first evaluated the robustness of scANMF under different types of noise. To enable controlled comparisons, we generated simulated datasets containing 1200 cells (4 cell types, 20 marker genes, 100 background genes). We evaluated the annotation accuracy under different scenarios: (i) incomplete prior information with 10%, 20%, or 30% of labels retained; (ii) noisy prior information with label error rates of 20%, 40%, or 60%; and (iii) noisy prior information with marker gene error rates of 20%, 40%, or 60%. The accuracy curves in Figure 4 show that scANMF consistently maintained a high annotation quality and a small variance across all settings. Under weak supervision, the accuracy remained stable when available labels were scarce. When noise was introduced into the prior knowledge, the model demonstrated a strong robustness, stayed above 0.95 under moderate noise, and remained reliable even with 60% noise.

#### 2.3.2. Parameter Sensitivity Analysis

**Figure 5:**
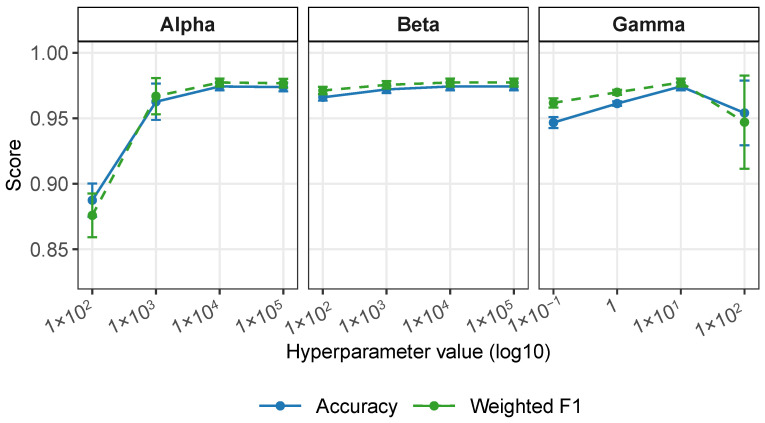
Parameter sensitivity analysis. scANMF maintained a high accuracy across wide parameter ranges for α, β, and γ.

We further evaluated the robustness of scANMF with respect to the three regularization parameters α, β, and γ on the Segerstolpe dataset. As shown in Figure 5, scANMF maintained a consistently high accuracy and weighted F1 across a wide range of parameter values. For α (marker constrain weight), the performance improved markedly when increasing α from $10^{2}$ to $10^{3}$, after which both the accuracy and the weighted F1-score plateaued near their maximum values and remained stable up to $10^{5}$. For β (label supervision weight), both metrics showed only minor fluctuations across four orders of magnitude. For γ (graph regularization weight), the performance peaked around γ=10, while both very small (0.1) and very large (100) values yielded slightly lower scores and an increased variance. Taken together, these results demonstrate that scANMF was robust to hyperparameter choices on the Segerstolpe dataset, maintaining a strong performance across wide regions of the parameter space. A parallel analysis using the Lawlor dataset, presented in Supplementary Figure S2, further confirms the stability of scANMF under diverse hyperparameter settings.

#### 2.3.3. Ablation Studies

**Figure 6:**
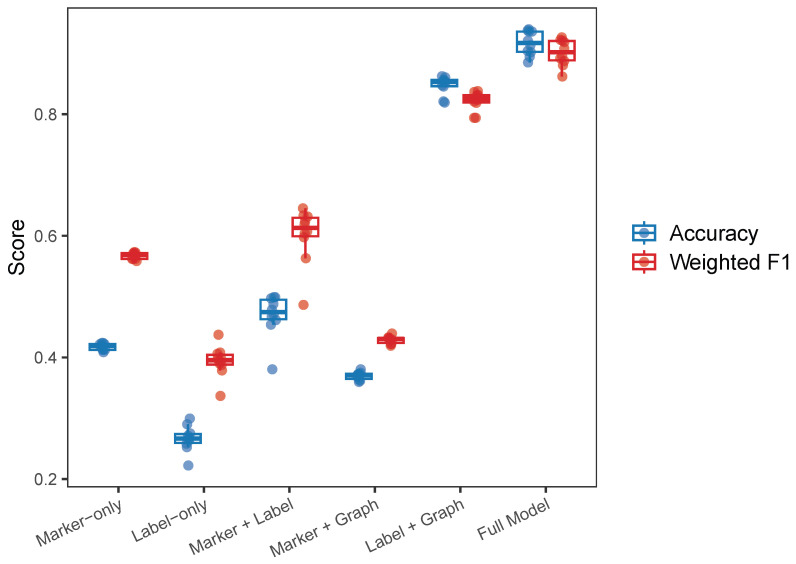
Ablation study results. The full model achieved the highest and most stable performance, and removing one or more terms led to performance degradation.

Ablation analyses were conducted using six model variants by combining or omitting marker constraints, label supervision, and graph regularization. Experiments were performed on the Lawlor dataset (Figure 6).

The full model achieved the highest accuracy and weighted F1-score across all ten runs, with minimal variance. The marker-only model performed substantially better than the label-only model, yielding a higher accuracy and a lower variability. When markers and labels were combined (marker + label), the performance improved beyond either source individually. Among the partial variants, label + graph achieved the best overall performance, highlighting that graph regularization effectively strengthens label supervision under the condition of limited labels. In contrast, marker + graph performed slightly worse than marker-only, suggesting that graph smoothness offers a limited benefit when markers are the only source of supervision. The models involving labels exhibited a greater variability across runs because the specific set of labeled cells changed each time, which in turn affected the quality of the supervision signal. Overall, the ablation study shows that, while all three components contribute positively, label supervision combined with graph regularization forms the core driver of performance, and marker constraints act as a complementary prior that further stabilizes and enhances the annotation accuracy when integrated with label information.

We also assessed the computational efficiency of scANMF. The runtime comparison with other annotation methods is provided in Supplementary Table S2.

## 3. Discussion

Accurate cell type annotation remains a central challenge in single-cell RNA sequencing analyses due to the sparsity, high dimensionality, and technical variability of gene expression data. Many existing methods rely primarily on a single source of prior information—such as marker genes or annotated reference datasets—which limits their robustness in settings where prior knowledge is incomplete, dataset-specific, or inconsistent across platforms or species. To address these issues, we developed scANMF, a prior- and graph-regularized non-negative matrix factorization framework that integrates marker-gene constraints, sparse label supervision, and the local manifold structure.

Several recently proposed annotation methods, including graph neural network-based models and atlas-driven integration pipelines, were not included in the quantitative benchmarking of this study. Many of these approaches rely on substantially different assumptions, making a direct comparison under a unified experimental protocol nontrivial. Accordingly, we focused on a representative set of widely used marker-based and reference-based methods that can be evaluated within a common framework. As a result, this study emphasizes the robustness and interpretability of scANMF under heterogeneous and partially noisy priors, rather than providing a comprehensive performance ranking across all existing tools.

Across a broad set of real-data evaluations, scANMF showed a consistently strong annotation performance under within-dataset, cross-platform, and cross-species conditions. Compared with scCATCH, ScType, scPred, and SingleR, scANMF achieved a higher accuracy and more stable results across different biological systems, sequencing technologies, and species. These observations suggest that jointly incorporating multiple forms of prior knowledge improves the model robustness when either the marker information or the labels alone are insufficient. In addition, the latent factors recovered by scANMF exhibited clear correspondence with cell-type-specific marker structures, indicating that the model preserves biologically meaningful patterns while performing annotation.

The robustness analyses further demonstrated that scANMF maintains a high performance under substantial noise in labels or marker genes and is insensitive to wide variations in hyperparameter choices. Ablation experiments confirmed that each of the three components contributes positively to the model performance and that the full model, integrating all sources of prior information, yields the most accurate and stable results.

The general strategy used in scANMF is consistent with prior studies emphasizing the benefits of integrating biological knowledge with machine learning frameworks. Unlike clustering- or reference-based methods, scANMF provides a unified factorization model that accommodates heterogeneous priors and local graph structure. This integrative design may be adaptable to related problems such as spatial transcriptomics and multimodal data integration analyses through modified regularization terms or coupled factorizations. However, despite these advantages, several methodological limitations remain that point toward future improvements.

First, scANMF requires users to specify three hyperparameters (α,β,γ), whose effective strengths depend on the scale of the normalized expression matrix and the density of the KNN graph. Although our sensitivity analyses showed a broad robustness across wide ranges of values, extreme mis-specification can still lead to a degraded performance in certain scenarios. For example, an overly large α or β may cause the model to overfit marker or label priors, suppressing data-driven structure, whereas an excessively small value can render these priors ineffective. Similarly, an ill-chosen γ may oversmooth the latent representation on dense graphs or fail to enforce manifold consistency on sparse graphs. Future work may explore data-driven strategies—such as stability-based tuning or Bayesian approaches that estimate the uncertainty over hyperparameters—to mitigate such sensitivity and enhance the robustness under minimal user tuning.

Second, the current formulation assumed that marker priors, label supervision, and the graph structure contribute additively and independently to the objective. Yet, in biological systems, these signals can be correlated. Explicitly modeling such interactions through hierarchical constraints or multiplicative coupling mechanisms may further improve the annotation fidelity.

Finally, as single-cell technologies continue to scale, contemporary cell atlases are increasingly encompassing datasets of a substantially larger size, posing additional computational challenges. In the current implementation, the cell–cell distances are computed directly in the preprocessed expression space after HVG selection and gene-wise standardization, which preserves fine-grained transcriptomic variation at moderate dataset scales. For larger-scale applications, the scalability could be further improved by constructing the KNN graph in a low-dimensional embedding, such as the PCA space, prior to graph regularization. Combined with sparse graph construction, mini-batch optimization, and distributed factorization, these extensions could facilitate the application of scANMF at the atlas scale.

In summary, scANMF provides an efficient, interpretable, and robust framework for cell-type annotation in scRNA-seq data. By jointly leveraging marker genes, partial labels, and the graph structure, the method performs reliably across heterogeneous datasets and maintains biological coherence in its latent representations, making it well suited for real-world applications where supervision is sparse or noisy.

## 4. Materials and Methods

### 4.1. Prior Knowledge and Graph-Regularized Non-Negative Matrix Factorization

Let $X=\left[X_{1},\ldots ,X_{n}\right]\in R_{\geq 0}^{m\times n}$ denote the gene-by-cell expression matrix, where each column $X_{j}$ represents the expression profile of cell *j* across *m* genes. Non-negative matrix factorization (NMF) approximates X with two non-negative matrices $U\in R_{\geq 0}^{m\times p}$ and $V\in R_{\geq 0}^{n\times p}$ by minimizing the reconstruction error:

(1) $$O_{1}={∥X-UV^{T}∥}_{F}^{2},\;U\geq 0,\;V\geq 0.$$

The matrix U contains gene–factor loadings and V contains the corresponding cell–factor coefficients. The factor number *p* was set to the cardinality of the union of cell types present in the marker and label priors.

Marker-Gene Regularization

Let $M^{0}\in R^{c\times p}$ encode marker-gene priors with

(2) $$M_{ik}^{0}=\left\{\begin{array}{cc} 1, & i\in G_{k},\\ 0, & \mathrm{otherwise}, \end{array}\right.$$

where $G_{k}$ denotes the marker set for cell type *k* and *c* is the number of marker genes. To match dimensions with U, the constraint matrix is expanded to

(3) $$M=\left(\begin{array}{c} M^{0}\\ 1_{(m-c)\times p} \end{array}\right),$$

where $1_{(m-c)\times p}$ ensures that the non-marker genes are unconstrained. Marker consistency is encouraged through an $l_{1}$ penalty:

(4) $$O_{2}=∥X-UV^{T}{∥}_{F}^{2}+\alpha {∥U⊙\left(1-M\right)∥}_{1},$$

where α controls the penalty strength, ⊙ denotes the Hadamard product, and 1 is an m×p all-ones matrix.

Label Supervision

Assume that the first *l* of *n* cells is annotated. The label constraint matrix $P^{0}\in R^{l\times p}$ is defined by

(5) $$P_{jk}^{0}=\left\{\begin{array}{cc} 1, & \mathrm{cell}\;j\;\mathrm{is}\;\mathrm{labeled}\;\mathrm{as}\;\mathrm{type}\;k,\\ 0, & \mathrm{otherwise}. \end{array}\right.$$

The full constraint matrix is

(6) $$P=\left(\begin{array}{c} P^{0}\\ 1_{(n-l)\times p} \end{array}\right).$$

Adding label supervision yields

(7) $$O_{3}=∥X-UV^{T}{∥}_{F}^{2}{+\alpha ∥U⊙\left(1-M\right)∥}_{1}+\beta {∥V⊙\left(1-P\right)∥}_{1},$$

where β regulates the label penalty.

Graph Regularization

To incorporate local cell–cell relationships, a mutual *K*-nearest-neighbor graph was constructed using Euclidean distances computed from the preprocessed expression matrix described in Section 4.5. Edge weights were computed using a Gaussian kernel,

(8) $$w_{j_{1}j_{2}}=\exp\;\left(-\frac{∥X_{j_{1}}-X_{j_{2}}{∥}^{2}}{2\sigma^{2}}\right),$$

where σ is the median pairwise distance of all KNN edge distances across the dataset. Let W denote the affinity matrix, D the degree matrix, and L=D−W the unnormalized graph Laplacian. The regularization term is

(9) $$\mathrm{Tr}\left(V^{T}LV\right)=\frac{1}{2}\sum_{j_{1},j_{2}}w_{j_{1}j_{2}}{∥V_{j_{1}}-V_{j_{2}}∥}^{2}.$$

Final Objective

An additional sparsity term on U was included to stabilize the scale. The complete objective is

(10) $$\begin{array}{cc} O_{F}= & ∥X-UV^{T}{∥}_{F}^{2}+\alpha_{0}{∥U∥}_{1}{+\alpha ∥U⊙\left(1-M\right)∥}_{1}+\beta {∥V⊙\left(1-P\right)∥}_{1}\\ \; & +\gamma \;\mathrm{Tr}(V^{T}LV),\;U\geq 0,\;V\geq 0, \end{array}$$

where $\alpha_{0}$ controls the sparsity and γ controls graph regularization.

### 4.2. Optimization of scANMF

Because $O_{F}$ is not jointly convex in (U,V), multiplicative update rules were derived from the Lagrangian and Karush–Kuhn–Tucker (KKT) conditions. Expanding the objective gives

(11) $$\begin{array}{cc} O_{F}= & \mathrm{Tr}\left(XX^{T}\right)-2\mathrm{Tr}\left(XUV^{T}\right)+\mathrm{Tr}\left(UV^{T}VU^{T}\right)+\alpha_{0}{∥U∥}_{1}\\ \; & {+\alpha ∥U⊙\left(1-M\right)∥}_{1}+\beta {∥V⊙\left(1-P\right)∥}_{1}+\gamma \;\mathrm{Tr}\left(V^{T}LV\right). \end{array}$$

Introducing multipliers Ψ and Φ for non-negativity constraints yields the Lagrangian

(12) $$\begin{array}{cc} L= & O_{F}+\mathrm{Tr}\left(\Psi U^{T}\right)+\mathrm{Tr}\left(\Phi V^{T}\right). \end{array}$$

The partial derivatives are

(13) $$\begin{array}{cc} \frac{\partial L}{\partial U} & =-2XV+2UV^{T}V+\alpha_{0}1+\alpha \left(1-M\right)+\Psi ,\\ \frac{\partial L}{\partial V} & =-2X^{T}U+2VU^{T}U+\beta \left(1-P\right)+2\gamma LV+\Phi . \end{array}$$

Applying the KKT complementarity conditions $\psi_{ik}u_{ik}=0$ and $\phi_{jk}v_{jk}=0$ results in the multiplicative updates

(14) $$\begin{array}{cc} u_{ik}\leftarrow u_{ik} & \;\frac{{\left(2XV\right)}_{ik}}{{\left(2UV^{T}V+\alpha_{0}1+\alpha \left(1-M\right)\right)}_{ik}},\\ v_{jk}\leftarrow v_{jk} & \;\frac{2{\left(X^{T}U+\gamma WV\right)}_{jk}}{{\left(2VU^{T}U+\beta \left(1-P\right)+2\gamma DV\right)}_{jk}}. \end{array}$$

Both U and V were initialized with non-negative random values drawn from a uniform distribution on [0,1]. NNDSVD initialization was evaluated, but it did not improve the performance, and random initialization was therefore used. The optimization iterated until the relative change in the objective function between two consecutive iterations satisfied

(15) $$\begin{array}{c} \frac{\left|O_{F}^{\left(t\right)}-O_{F}^{\left(t-1\right)}\right|}{O_{F}^{\left(t-1\right)}}<10^{-4}, \end{array}$$

or when the maximum number of iterations (50) was reached.

The final annotation for cell *j* was determined by the index of the maximum element in row $V_{j}$.

### 4.3. Data Simulation

To evaluate the annotation models under controlled conditions, we developed a single-cell expression simulation framework incorporating background variation, the marker-driven structure, dropout, heteroscedastic noise, and batch effects. Genes were divided into shared (background) and differential (marker-associated) components. Shared gene means were drawn from

(16) $$m_{i}∼N\left(\mu_{0},\sigma_{0}^{2}\right).$$

For each cell type *k* with marker set $G_{k}$, the type-specific mean of gene *i* was defined as

(17) $$\mu_{ik}=\left\{\begin{array}{cc} a\;m_{i}, & i\in G_{k},\\ m_{i}-\delta , & \mathrm{otherwise}, \end{array}\right.\;a>1,\;\delta >0.$$

Dropout was simulated by setting entries to zero with a probability $\pi_{0}\in \left[0,1\right]$. The observed expression of gene *i* in cell *j* with type c(j) was modeled as

(18) $$X_{ij}=\mu_{i,c(j)}+\varepsilon_{ij},\;\varepsilon_{ij}∼N\;\left(0,{\left(\sigma_{\mathrm{hetero}}\mu_{i,c(j)}+\sigma_{\mathrm{homo}}\right)}^{2}\right),$$

where $\sigma_{\mathrm{hetero}}$ and $\sigma_{\mathrm{homo}}$ represent the heteroscedastic and homoscedastic noise components. Negative values were truncated at zero. Batch effects were introduced by applying fixed shifts to the gene means across predefined groups. This procedure generates matrices that exhibit key characteristics of the scRNA-seq data, including the noise, sparsity, and batch-level variation.

### 4.4. Real-Data Collection

Publicly available scRNA-seq datasets were compiled from GEO and ArrayExpress to evaluate the within-dataset, cross-platform, and cross-species annotation performance.

#### 4.4.1. Intra-Dataset Annotation

Six benchmark datasets were used, consisting of three brain and three pancreas datasets (Table 5). To mimic realistic scenarios where only a small number of cell labels are available, only small fractions of labels were retained for each dataset: 10% for Darmanis, 5% for Lawlor, and 2% for all remaining datasets. Labeled cells were randomly sampled, and all methods requiring labels received the same sets. Each experiment was repeated ten times to account for variability. For scCATCH and ScType, which do not rely on label input, the results were computed directly on the unlabeled portion of each dataset.

#### 4.4.2. Cross-Platform Annotation

Cross-platform evaluations were performed using the mouse brain datasets (Zeisel and Romanov) generated with STRT-Seq UMI and Smart-Seq2, respectively, and multiple human pancreas datasets produced by inDrop, CEL-Seq2, SMARTer, and Smart-Seq2. Genes shared across datasets were retained, resulting in 14,818 cells and 3000 genes. The data were grouped into two platform sets, as summarized in Table 6.

#### 4.4.3. Cross-Species Annotation

Cross-species experiments were conducted between human and mouse brain datasets (Zeisel, Romanov, Darmanis) and Baron human–mouse pancreas data (Table 7), which enabled the isolation of species differences while controlling for platform variability. To mitigate the dataset size imbalance between the Baron human and mouse pancreas data and the improve computational efficiency, we randomly sampled 20% of the human cells when constructing the training set.

### 4.5. Real-Data Preprocessing

Cells expressing fewer than 200 genes or with over 20% mitochondrial transcripts were removed. Genes expressed in at least three cells were retained. Counts $x_{ij}^{0}$ were normalized by library size $s_{j}=\sum_{i}x_{ij}^{0}$, scaled to $10^{4}$, and log-transformed:

(19) $$x_{ij}^{\prime }=\log\left(\frac{x_{ij}^{0}}{s_{j}}\times 10^{4}+1\right).$$

Highly variable genes (HVGs) were selected using mean–variance modeling, and the top 1000 by standardized variance $v_{j}$ were used:

(20) $$v_{j}=\frac{\sigma_{j}^{2}}{f(\mu_{j})},$$

where $f(\mu_{j})$ denotes the fitted mean–variance trend, representing the expected variance of a gene with average expression $\mu_{j}$, following the standard approaches used in Seurat for HVG selection.

Gene-wise standardization was applied:

(21) $$x_{ij}=\frac{x_{ij}^{\prime }-\mu_{i}}{\sigma_{i}}.$$

Curated marker genes were obtained from ScType. Genes not present in the expression matrix were excluded. The final matrix consisted of the union of HVGs and marker genes. A binary marker matrix encoded gene–cell-type relationships.

### 4.6. Regularization Parameter Search

Hyperparameters α, β, and γ were tuned separately for the within- and cross-dataset experiments. For within-dataset annotation, α=10,000, β=10,000, and γ=10 were applied. For the cross-dataset settings, a two-stage search was performed over γ∈{0.1,1,10,100} and $\alpha ,\beta \in \{10^{2},10^{3},10^{4},10^{5}\}$, followed by fine-scale refinement around high-performing combinations. The five-fold cross-validation accuracy was used for selection. The full parameter configurations are listed in Supplementary Table S1. In addition, $\alpha_{0}$ was fixed at 50. The number of neighbors *K* in the mutual KNN graph was selected based on the dataset size. For smaller datasets, we set *K* to approximately one-third of the total number of cells. For larger datasets, we capped *K* at 1000 to control the graph density and computational cost. Accordingly, the values of *K* used in our experiments fell within {100,300,1000}.

### 4.7. Benchmark Methods

Four representative annotation tools were included:

- **scCATCH (v3.2.2)** [21]: cluster-level annotation using CellMatch marker references.
- **ScType** [16]: marker-based cluster annotation with integrated positive/negative marker sets.
- **SingleR (v2.8.0)** [32]: reference-based cell-level annotation using correlation with reference profiles.
- **scPred (v1.9.2)** [34]: supervised cell-level classifier trained on reduced representations.

All of the methods were run with the recommended default parameters unless specified. When label subsets were required, identical label sets were provided to all the applicable methods.

### 4.8. Evaluation Metrics

Classification-based evaluation metrics

The accuracy was computed as

(22) $$\mathrm{Accuracy}=\frac{n_{\mathrm{correct}}}{n_{\mathrm{all}}}\times 100\%,$$

where $n_{\mathrm{correct}}$ is the number of correctly annotated cells and $n_{\mathrm{all}}$ is the total number of cells. Moreover, the precision and recall were defined as

(23) $$\begin{array}{cc} \mathrm{Precision} & =\frac{\mathrm{TP}}{\mathrm{TP}+\mathrm{FP}},\\ \mathrm{Recall} & =\frac{\mathrm{TP}}{\mathrm{TP}+\mathrm{FN}}, \end{array}$$

where TP, FP, and FN denote true positives, false positives, and false negatives, respectively.

Since the precision and recall highlight complementary aspects of performance, their harmonic mean, the F1-score, was also considered:

(24) $$F1-\mathrm{score}=2\times \frac{\mathrm{Precision}\times \mathrm{Recall}}{\mathrm{Precision}+\mathrm{Recall}}.$$

Finally, in multi-class settings, the weighted F1-score was calculated to mitigate class imbalance by accounting for the number of samples in each class:

(25) $$\mathrm{weighted}\;F1-\mathrm{score}=\frac{\sum_{i=1}^{K}{F1-\mathrm{score}}_{i}\cdot n_{i}}{\sum_{i=1}^{K}n_{i}},$$

where *K* is the total number of classes, ${F1-\mathrm{score}}_{i}$ is the F1-score of class *i*, and $n_{i}$ is the number of samples in class *i*.

Consistency Analysis with Marker-Gene Priors

To assess whether the latent factors learned by the NMF model captured the biologically meaningful cell-type structure, we evaluated the agreement between U and M.

For each marker gene *i*, the factor on which it achieves maximal loading was defined as

(26) $$k^{*}\left(i\right)=\arg\max_{1\leq k\leq p}U_{ik}.$$

Let $C_{i}=\left\{\;c|M_{ic}=1\;\right\}$ denote the annotated cell type set of gene *i*. To properly accommodate many-to-many relationships between marker genes and cell types, each marker assignment was weighted by

(27) $$w_{ic}=\left\{\begin{array}{cc} \frac{1}{|C_{i}|}, & c\in C_{i},\\ 0, & \mathrm{otherwise}. \end{array}\right.$$

The number of marker genes from cell type *c* whose dominant factor is *k* is

(28) $$n_{c\to k}=\left|\{\;i\in G_{c}^{\mathrm{marker}}|k^{*}\left(i\right)=k\;\}\right|.$$

Normalizing over all marker genes of cell type *c* yields the cell-type-normalized marker-gene proportion,

(29) $$P_{c\to k}=\frac{n_{c\to k}}{|G_{c}^{\mathrm{marker}}|},$$

which quantifies the fraction of marker genes of cell type *c* assigned to latent factor *k*.

A marker gene is considered correctly assigned if the cell type associated with its dominant factor matches any of its annotated cell types. The factor *k* was assigned to the cell type $c^{*}\left(k\right)=\arg\max_{c}n_{kc}$. The resulting marker-gene accuracy is

(30) $${\mathrm{Accuracy}}_{\mathrm{marker}}=\frac{\sum_{i:C_{i}\neq ⌀}1\left\{c^{*}\left(k^{*}\left(i\right)\right)\in C_{i}\right\}}{\left|\{\;i:C_{i}\neq ⌀\;\}\right|}.$$

Together, these two metrics provide complementary gene-level and cell-type-level assessments of how well the latent factors align with the known marker-gene structure.

## 5. Conclusions

In this study, we introduced scANMF, a graph-regularized non-negative matrix factorization framework for accurate and robust cell-type annotations in single-cell RNA sequencing data. By integrating marker-gene information, partial label supervision, and the local cell–cell graph structure within a unified optimization objective, scANMF effectively balances the interpretability and predictive performance under heterogeneous and weakly supervised settings. Extensive evaluations across within-dataset, cross-platform, and cross-species scenarios demonstrated that scANMF consistently achieves a high accuracy and a stable performance, particularly in the presence of incomplete or noisy prior knowledge. Moreover, the learned latent factors exhibited strong biological coherence, providing transparent links between inferred cell types and the known marker-gene structure. Together, these results highlight scANMF as a practical and interpretable annotation framework with a broad applicability to real-world single-cell studies where supervision is limited or imperfect.

## Figures and Tables

**Table 1:** Cross-platform annotation performance in two directions: (1) Zeisel → Romanov and (2) Romanov → Zeisel.

Method	Zeisel → Romanov		Romanov → Zeisel	
	Accuracy	Weighted F1-Score	Accuracy	Weighted F1-Score
scANMF	**88.27%**	**0.8692**	**95.11%**	**0.9481**
scCATCH	56.23%	0.6873	51.01%	0.6498
ScType	75.81%	0.7516	87.25%	0.8817
scPred	30.96%	0.1510	53.78%	0.4185
SingleR	78.38%	0.7244	94.64%	0.9553

**Table 2:** Cross-platform annotation performance in two directions: (1) Pancreas 1: Train: Baron+Muraro → Test: Xin+Segerstolpe+Lawlor and (2) Pancreas 2: Train: Xin+Segerstolpe+Lawlor → Test: Baron+Muraro.

Method	Pancreas 1		Pancreas 2	
	Accuracy	Weighted F1-Score	Accuracy	Weighted F1-Score
scANMF	**98.32%**	**0.9838**	**95.18%**	**0.9492**
scCATCH	80.19%	0.8184	63.06%	0.6442
ScType	93.72%	0.9238	90.59%	0.9014
scPred	33.24%	0.4745	47.80%	0.6188
SingleR	96.97%	0.9704	94.93%	0.9461

**Table 3:** Cross-species annotation performance on human and mouse brain datasets.

Method	Zeisel → Darmanis		Darmanis → Zeisel		Romanov → Darmanis		Darmanis → Romanov	
	Accuracy	Weighted F1-Score	Accuracy	Weighted F1-Score	Accuracy	Weighted F1-Score	Accuracy	Weighted F1-Score
scANMF	**91.93%**	**0.9027**	**93.81%**	**0.9396**	**91.22%**	0.8846	**76.40%**	0.7208
scCATCH	69.82%	0.7521	51.01%	0.6498	69.82%	0.7521	56.23%	0.6873
ScType	80.00%	0.8251	87.25%	0.8817	80.00%	0.8251	75.81%	**0.7516**
scPred	39.30%	0.3300	56.77%	0.4306	86.67%	**0.8871**	73.03%	0.6809
SingleR	90.18%	0.8741	91.88%	0.9155	88.42%	0.8620	74.07%	0.6860

**Table 4:** Cross-species annotation performance in two directions: (1) Baron_human → Baron_mouse and (2) Baron_mouse → Baron_human.

Method	Baron_human → Baron_mouse		Baron_mouse → Baron_human	
	Accuracy	Weighted F1-Score	Accuracy	Weighted F1-Score
scANMF	**93.79%**	**0.9475**	**95.85%**	**0.9594**
scCATCH	27.36%	0.3318	67.20%	0.6608
ScType	93.63%	0.9288	92.13%	0.9081
scPred	89.78%	0.9106	62.38%	0.6389
SingleR	80.19%	0.8134	49.43%	0.3827

**Table 5:** Summary of scRNA-seq datasets used for intra-dataset annotation.

Dataset	Protocol	Species	Tissue	Total Cells	Genes	Cell Types
Darmanis [51] (GSE84465)	SMARTer	Human	Brain	466	22,085	9
Zeisel [52] (GSE60361)	STRT-Seq UMI	Mouse	Brain	3005	20,006	7
Romanov [53] (GSE74672)	Smart-Seq2	Mouse	Brain	2881	24,341	7
Lawlor [54] (GSE86473)	SMARTer	Human	Pancreas	638	26,616	8
Muraro [55] (GSE85241)	CEL-Seq2	Human	Pancreas	3072	19,059	11
Segerstolpe [56] (E-MTAB-5061)	Smart-Seq2	Human	Pancreas	3514	26,179	15

**Table 6:** Summary of human pancreatic scRNA-seq datasets used for cross-dataset annotation.

Dataset	Protocol	Cells After Processing	Genes After Processing	Cell Types
Baron [57] (GSE84133)	inDrop	10,600	3000	14
Muraro [55] (GSE85241)	CEL-Seq2			
Xin [58] (GSE81608)	Smart-seq2			
Segerstolpe [56] (E-MTAB-5061)	Smart-seq2	4218	3000	11
Lawlor [54] (GSE86473)	SMARTer			

**Table 7:** Summary of human and mouse pancreatic scRNA-seq datasets used for cross-species annotation.

Dataset	Protocol	Species	Tissue	Total Cells	Genes	Cell Types
Baron [57] (GSE84133)	inDrop	Human	Pancreas	8569	20,125	14
Baron [57] (GSE84133)	inDrop	Mouse	Pancreas	1886	14,878	13

## Data Availability

All the datasets analyzed in this study are publicly available. Specifically, the single-cell RNA sequencing (scRNA-seq) datasets were obtained from the Gene Expression Omnibus (GEO, https://www.ncbi.nlm.nih.gov/geo/; accessed on 17 December 2025) and ArrayExpress (https://www.ebi.ac.uk/arrayexpress/; accessed on 17 December 2025) under the following accession numbers: GSE67835, GSE81608, GSE85241, GSE84133, GSE86473, and E-MTAB-5061. No new datasets were generated in this work. The code for scANMF is available at https://github.com/klovbe/scANMF; accessed on 17 December 2025.

## Supplementary Materials

The following supporting information can be downloaded at: https://www.mdpi.com/article/10.3390/ijms27010125/s1.
