# Supplementary material for: scANMF: Prior Knowledge and Graph-Regularized NMF for Accurate Cell Type Annotation in scRNA-seq
Source: Int J Mol Sci. 2025 Dec 22;27(1):125. doi: 10.3390/ijms27010125 (PMC12785987; doi:10.3390/ijms27010125)
Supplement: Supplementary file 1 [file ijms-27-00125-s001.zip › ijms-4035562-supplementary.pdf]

Supplementary Materials

Overview

This Supplementary Materials document provides additional figures, parameter settings used across experiments, and extended analysis of computational runtime and cell-type–specific annotation performance. These materials support the results presented in the main manuscript and offer further insights into model behavior and evaluation.

1 Parameter Settings

The simulation parameters introduced in Section 3.3.1 of the main text are:

$$\mu = 1, \quad \sigma^2_{\text{homo}} = 0.1, \quad \sigma^2_{\text{hetero}} = 0.2, \quad a = 1.5, \quad \delta = 0.5.$$

The tuning parameters (α, β, γ) for all experiments are summarized in Table S1.

Table S1: Parameter settings for all experiments.

Section	Dataset	$\boldsymbol{\alpha}$	$\boldsymbol{\beta}$	$\boldsymbol{\gamma}$
3.1.1	All	10000	10000	10
3.1.2	Zeisel → Romanov	10000	10000	10
	Romanov → Zeisel	2000	1000	50
	Pancreas 1	10000	10000	10
	Pancreas 2	10000	10000	10
3.1.3	Zeisel → Darmanis	10000	10000	10
	Darmanis → Zeisel	2000	1000	10
	Romanov → Darmanis	1000	1000	10
	Darmanis → Romanov	1000	300	30
	Baron (Mouse) → Baron (Human)	1000	1000	10
	Baron (Human) → Baron (Mouse)	10^6	20	1
3.2	Noise	10	100	0.1

2 Supplementary Figures

2.1 Cross-Platform Factor–Marker Structure

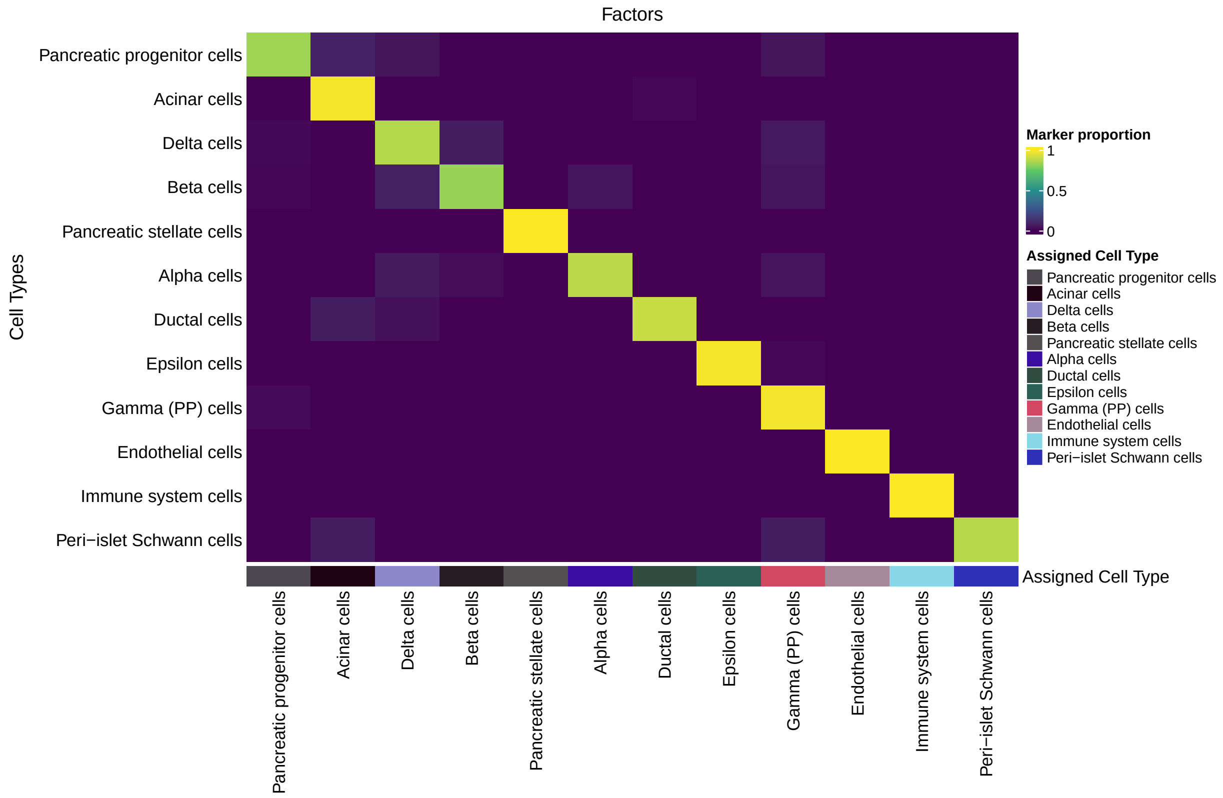

Figure S1: Cell-type–normalized marker-gene proportion matrix $P_{c \to k}$ in the cross-platform pancreas experiment. Each row corresponds to a cell type and each column to a latent factor. The strong diagonal pattern demonstrates that each latent factor concentrates marker genes corresponding to a specific cell type. The inferred factor identities (bottom color bar) match annotated cell types with perfect marker-gene mapping accuracy ($\text{Accuracy}_{\text{marker}} = 1.0$).

2.2 Parameter Sensitivity Analysis

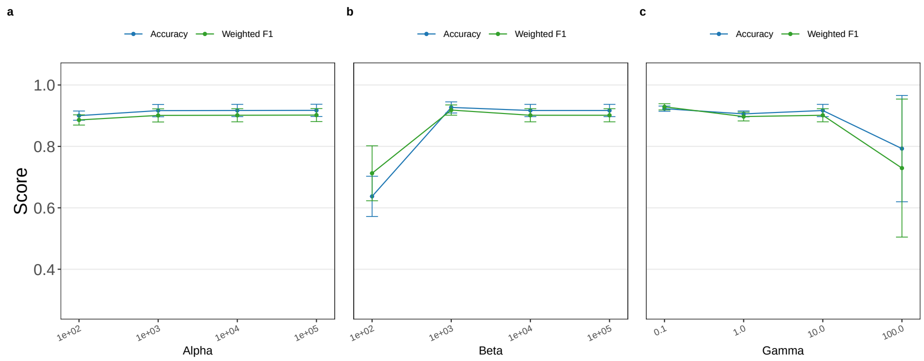

Figure S2: Parameter sensitivity analysis on the Lawlor dataset. scANMF maintains stable and high accuracy and weighted F1 across wide ranges of hyperparameters. (1) For α (marker constraint weight), performance remains stable from 10^2 to 10^5. (2) For β (label supervision weight), accuracy improves between 10^2 and 10^3 before plateauing at high performance. (3) For γ (graph regularization weight), performance peaks at $\gamma = 0.1$, remains stable up to $\gamma = 10$, and decreases at $\gamma = 100$.

3 Supplementary Note

3.1 Computational Runtime

Table S2 reports the runtime of all annotation methods across the three cross-dataset settings. The computational cost varies substantially due to differences in model structure and reliance on marker sets or reference data.

ScType, which relies solely on marker look-up and enrichment scoring, is consistently the fastest. Methods such as scPred and SingleR exhibit runtime increases as the training or reference set grows larger. scANMF shows moderate runtime dependence on both training and test set sizes, as its matrix factorization step jointly involves both components. Despite this, runtime remains well within a practical range. When moving from Zeisel to the larger Baron datasets, scANMF maintains good scalability and remains competitive with learning-based methods such as scPred. Furthermore, scANMF allows subsampling of the training dataset without compromising annotation performance, providing an effective mechanism for reducing computational cost on large datasets.

Table S2: Runtime comparison of annotation methods across three datasets.

**Method**	**Baron (Mouse)**	**Baron (Human)**	**Zeisel**
scANMF	516.661 s	345.752 s	43.057 s
scCATCH	42.084 s	150.957 s	1138.542 s
ScType	0.857 s	4.392 s	1.412 s
scPred	399.316 s	70.241 s	9.280 s
SingleR	119.586 s	104.998 s	1.147 s

3.2 Cell-type–specific annotation performance

To complement global performance metrics, we examined cell-type–specific annotation behavior using the confusion matrix on the Pancreatic 1 dataset (Table S3). This dataset exhibits pronounced variation in cell-type abundance, enabling evaluation of prediction patterns across both highly represented and sparsely represented populations.

Highly abundant endocrine populations, including Alpha and Beta cells, show strong diagonal dominance in the confusion matrix, indicating high overall annotation accuracy. Nevertheless, a small number of cells from these dominant classes are assigned to other categories, demonstrating that minor misclassifications are not restricted to low-abundance cell types.

Several sparsely represented populations, such as Endothelial, Mast, and Epsilon cells, achieve perfect or near-perfect recall, with all cells from these types correctly classified. However, due to their limited sample sizes, even a small number of cells from other types assigned to these categories results in a noticeable reduction in precision. This pattern is primarily driven by the limited sample sizes of these populations.

Table S3: Confusion matrix of scANMF annotation results on the pancreatic dataset. Rows correspond to true cell-type labels, and columns correspond to predicted labels.

**True \ Predicted**	Beta	Stellate	Ductal	Alpha	Acinar	Delta	Endothelial	Mast	Epsilon	Gamma (PP)	Immune	Schwann
Beta cells	**995**	0	0	2	0	1	3	3	0	0	1	1
Pancreatic stellate cells	0	**70**	1	0	0	0	2	0	0	0	0	0
Ductal cells	1	0	**411**	1	0	0	0	0	1	0	0	0
Alpha cells	0	0	0	**2002**	0	0	3	0	0	2	1	2
Acinar cells	0	0	31	1	**176**	0	0	1	0	0	0	0
Delta cells	0	0	2	3	0	**181**	1	1	0	0	0	0
Endothelial cells	0	0	0	0	0	0	**16**	0	0	0	0	0
Mast cells	0	0	0	0	0	0	0	**7**	0	0	0	0
Epsilon cells	0	0	0	0	0	0	0	0	**7**	0	0	0
Gamma (PP) cells	0	0	0	0	0	0	0	0	0	**282**	0	0

The most prominent confusion occurs between Acinar and Ductal cells, which are both represented at intermediate abundance. Misclassifications between these two cell types account for the largest off-diagonal entries in the confusion matrix, indicating that they constitute the primary source of annotation ambiguity in this dataset.

Overall, the confusion matrix reveals that scANMF maintains high sensitivity across cell types of varying abundance, while misclassifications are sparse and primarily concentrated among transcriptionally related populations. These cell-type–resolved results provide a more nuanced view of annotation performance beyond aggregate accuracy measures. These observations are further supported by low-dimensional visualizations highlighting misclassified cells in Figure S3.

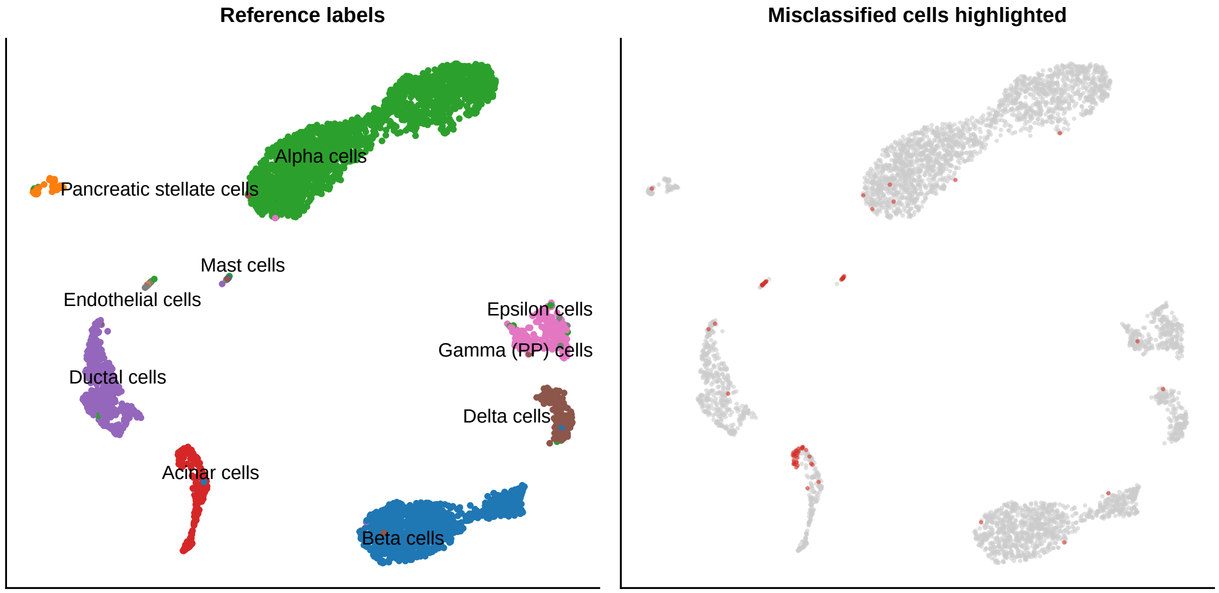

Figure S3: UMAP visualization based on the PCA-transformed processed expression space. Cells are colored by reference cell-type labels (left) and by misclassification status under scANMF (right), where misclassified cells are highlighted. Axes correspond to the first two UMAP dimensions.
